# Black Garlic: Evolution of the Chemical Composition and Broad Biological Activities

**DOI:** 10.1021/acs.jafc.5c09705

**Published:** 2025-10-22

**Authors:** Valentina Vecchi, Giuseppe Sabbioni, Maria Teresa Altieri, Giulia Breveglieri, Stefania Costa, Filippo Marchetti, Irene Gugel, Silvia Vertuani, Monica Borgatti

**Affiliations:** Department of Life Sciences and Biotechnology, 9299 University of Ferrara, Ferrara 44121, Italy

**Keywords:** black garlic, *Allium sativum* L., Maillard reaction, bioactive compounds, functional food

## Abstract

Black garlic, a product obtained from fresh garlic (*Allium sativum* L.) under specific temperature and humidity conditions, has garnered significant scientific attention due to its enhanced organoleptic qualities and superior health-promoting properties compared to the fresh product. The Maillard reaction, alongside other chemical transformations, converts pungent organosulfur compounds like allicin into stable, highly bioactive compounds such as *S*-allyl-l-cysteine and *S*-allylmercaptocysteine, while also increasing the levels of polyphenols and other potent antioxidants. This review provides a comprehensive overview of black garlic, detailing the impact of its unique ripening process on its chemical composition, encompassing changes in sugars, proteins, amino acids, lipids, vitamins, minerals, and organic acids. We explore how variations in temperature and humidity during production influence the final product quality attributes and bioactive compound profiles. Furthermore, we extensively discuss the diverse biological activities of black garlic and its key constituents, including significant antioxidant, anti-inflammatory, anticancer, immunostimulatory, antiallergic, hepatoprotective, antidiabetic, and antiobesity effects. By synthesizing current research, this review highlights black garlic potential as a functional food and a source of therapeutic agents, while also emphasizing the need for standardized production methods to ensure consistent quality and maximize its health benefits.

## Graphical abstract

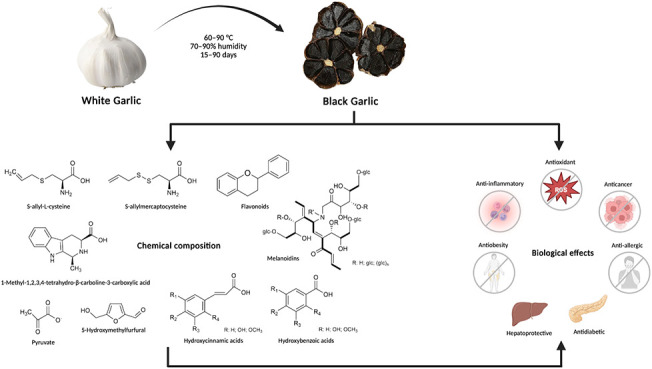

## 1 Introduction

In recent decades, scientific research has been increasingly focusing on investigating edible plants health-promoting benefits and potential therapeutic applications. These species are rich in phytochemicals that may help prevent or delay the onset of various diseases and provide valuable support in their treatment.^1^ Among these plants, garlic (*Allium sativum L.*) has garnered considerable attention, partly due to its long-standing use in traditional medicine for treating various ailments.^2^ Nevertheless, fresh garlic (FG) consumption has declined due to its strong flavor and pungent odor, along with the gastrointestinal discomfort it may cause in certain individuals.^3,4^ To address this issue, various garlic-based products have been developed to enhance its organoleptic attributes. Among the commercially available garlic-derived products, black garlic (BG) is one of the most studied.

Although the origin of BG remains unclear, historical evidence suggests that it has been consumed in Asian countries since ancient times.^5^ BG is obtained by aging fresh garlic bulbs at controlled high temperature (60–90 °C) and relative humidity (70–90%) for 15 to 90 days, without the use of any additives.^6,7^ During this transformation, garlic cloves acquire a dark color (Figure 1), a sweeter flavor, and a chewy texture.^1,8^ The color change results from various chemical transformations, including the Maillard reaction, caramelization, and the oxidation of phenols.^3^

**Figure 1:**
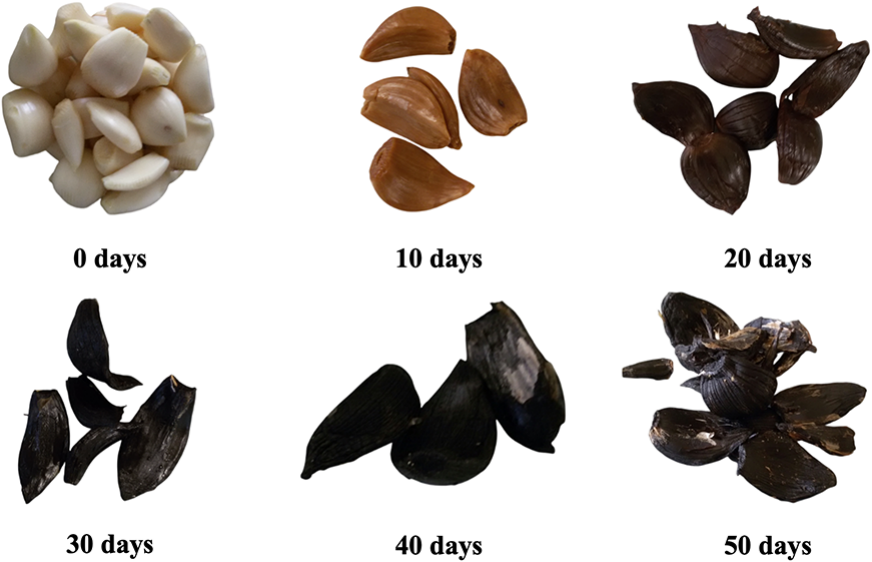
Changes in the color of black garlic during the ripening process.

In contrast, changes in flavor and texture are primarily associated with the accumulation of reducing sugars and the degradation of cell wall polysaccharides under high-temperature conditions, ultimately resulting in a loss of tissue hardness.^8^

During this process, molecules responsible for the distinctive aroma of FG, such as allicin, are converted into other compounds, including S-allyl-l-cysteine (SAC) and S-allylmercaptocysteine (SAMC), thereby reducing the unpleasant odor.^1^

The transformation of FG into BG not only modifies its organoleptic characteristics but also enhances the content of bioactive substances, as polyphenols and organosulfur compounds.^9^

Fresh garlic is widely recognized for its beneficial properties, including antibacterial,^10^ antiviral,^11^ antidiabetic,^12^ antioxidant,^13^ anti-inflammatory,^14^ antihypertensive,^15^ cardioprotective,^16^ hypolipidemic,^17^ and immunomodulatory^18^ effects.

Nevertheless, black garlic displays distinct biological activity that differs from that of fresh garlic. In particular, BG possesses higher antioxidant activity^19^ and exerts anti-inflammatory,^20^ anticancer,^21^ immunostimulatory,^22^ antiallergic,^23^ hepatoprotective,^24^ antidiabetic,^25^ and antiobesity^26^ effects.

Therefore, black garlic could be proposed as a functional food, offering health benefits that extend beyond basic nutrition when regularly included in a balanced diet and consumed in appropriate amounts.^27^

This review provides an overview of the main mechanisms underlying BG production and their impact on its physicochemical properties and bioactivity. Relevant studies were retrieved through targeted searches in PubMed and ScienceDirect, with particular attention to research addressing processing conditions, chemical transformations, and the biological activities of the resulting bioactive compounds. By integrating current evidence, this work aims to clarify the functional potential of black garlic and outline areas requiring further investigation.

## 2 Black Garlic Production

Currently, challenges persist in obtaining a product with consistent qualitative characteristics and biological effects, primarily due to the lack of a standardized manufacturing method and a comprehensive understanding of the desired compounds formation mechanisms, aside from the well-established involvement of the Maillard reaction.^3,4^

The Maillard reaction (Figure 2) is a nonenzymatic browning reaction that occurs between the carbonyl groups of reducing sugars and the amino groups of amino acids, peptides, and proteins. This process develops through three main stages. Initially, reducing sugars react with amino acids, leading to the formation of Amadori or Heyns products, depending on whether the sugar is an aldose or a ketose, respectively.^28^ Yuan et al. observed a 40- to 100-fold increase in the main Amadori and Heyns compounds in BG compared to FG.^7^ In the second stage, sugar fragmentation and amino acid degradation occur, resulting in the formation of various intermediates, such as 5-hydroxymethylfurfural (HMF). In the final phase, these compounds polymerize, leading to the production of high-molecular-weight brown polymers known as melanoidins.^28^

**Figure 2:**
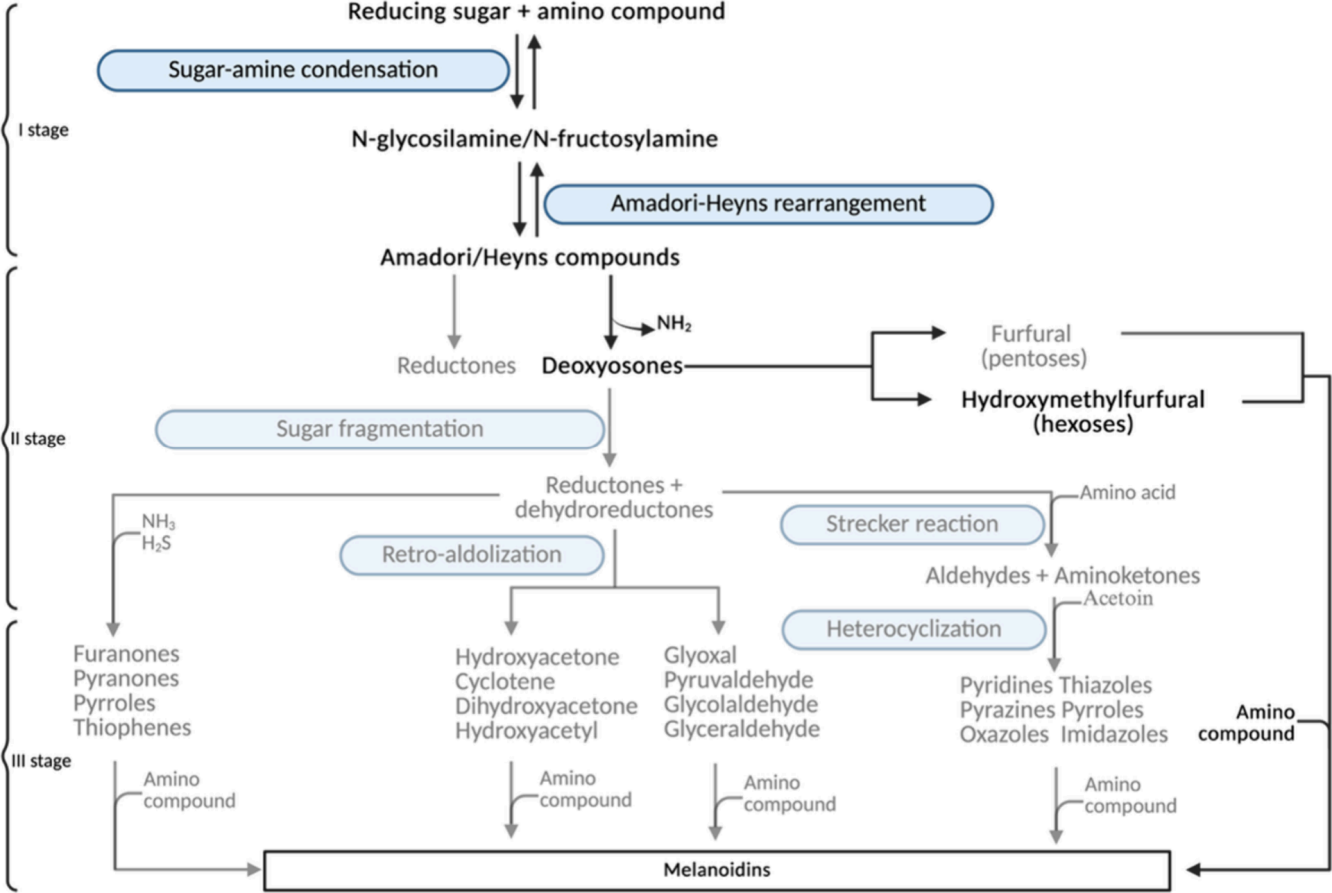
Schematic representation of the different stages of the Maillard reaction involved in black garlic ripening (modified from Yoon & Baek^29^). Created in BioRender. BORGATTI, M. (2025) https://BioRender.com/sz5f9z4.

The Maillard reaction contributes to changes in the nutritional profile, color, texture, and flavor of garlic.^2,30^

These transformations are strongly influenced by temperature and relative humidity, both of which play a decisive role in determining black garlic quality attributes.^4,31^ Higher temperatures accelerate the ripening process and intensify the final products’ color and flavor, but excessive heat (e.g., 90 °C) can lead to a bitter taste due to the rapid depletion of reducing sugars, which are consumed to sustain the Maillard reaction. Instead, humidity critically determines the product texture, with optimal conditions achieved when the water content reaches 400–500 g/kg. Conversely, when it falls below 350 g/kg, BG becomes too hard to be consumed.^31^

Processing conditions also alter the concentration of bioactive compounds in black garlic.^4,32^ For example, subjecting fresh garlic to a temperature of 60 °C enhances the levels of SAC, the primary antioxidant compound in BG.^33^ However, the accumulation of HMF, another significant antioxidant molecule, occurs at a considerably slower rate at this temperature.^31^ HMF production also depends on the duration of the ripening period; indeed, its concentration increases more than 6-fold when the period has been extended from 25 to 90 days.^34^

A detailed overview of the mechanisms behind BG ripening could help identify optimal production conditions to enhance organoleptic properties, nutritional value, and bioactivity.^6^

Although the changes underlying BG production are largely attributed to nonenzymatic reactions driven by heat and humidity, emerging evidence suggests that endophytic microorganisms may also contribute to the ripening process. Only a few studies have examined the microbial species found in garlic, which primarily belong to the *Bacillus* genus, a bacterial strain commonly found in soil, water sources and plants. More specifically, Qiu and colleagues isolated 78 endophytic strains during black garlic processing and found that *Bacillus subtilis* remained dominant throughout, with *B. methylotrophicus* and *B. amyloliquefaciens* also contributing significantly to the microbial community.^35^ Additionally, bacteria from the genera *Thermus*, *Corynebacterium*, *Streptococcus*, and *Brevundimonas* have been identified.^36^

These microorganisms can adapt to various carbon sources and exhibit significant heat resistance. Therefore, they could play a role in the development of compounds that contribute to the flavor and bioactivity of BG.^35,36^

In a subsequent study, Qiu et al. selected the most relevant endophytes identified in black garlic, based on their relative abundance and preliminary experimental findings, to examine their contributions during the aging process. The investigation involved four *B.* strains, including the three previously mentioned, with the addition of *B. licheniformis*, and confirmed their ability to proliferate across a broad temperature range (20–50 °C) and pH spectrum (5–9). Notably, when the temperature reaches 50 °C, the growth of both *B. subtilis* and *B. amyloliquefaciens* undergo a marked decline, whereas *B. licheniformis* and *B. methylotrophicus* growth appear less sensitive. Moreover, the inoculation of the four strains with garlic polysaccharide and garlic juice media demonstrated their capacity to hydrolyze garlic polysaccharides, thereby increasing the percentage of reducing sugars. Finally, by inoculating the endophytes with fresh garlic cloves, the authors illustrated that, in comparison to controls, *B. methylotrophicus*, *B. amyloliquefaciens*, and *B. subtilis* can slightly accelerate the formation of black garlic (0.8–2.8%), in contrast to *B. licheniformis*, which delays the browning process. Collectively, these findings underscore the potential impact of endophytes on aging dynamics, although further research is necessary to provide deeper insights into this phenomenon.^37^

In addition to ripening conditions and microbial influences, the intrinsic characteristics of fresh garlic contribute significantly to the physicochemical and bioactive properties of the final product. In particular, garlic variety affects moisture content, polyphenol concentration, total soluble solids, pH, antioxidant activity, texture, and color. Nevertheless, most fresh garlic traits are not reliable predictors of BG quality. Consequently, additional studies are required to clarify which specific attributes of fresh garlic are decisive in determining the final characteristics of black garlic.^38,39^

## 3 Comparison of the Chemical Composition of Fresh and Black Garlic

The chemical profile of fresh garlic changes significantly due to several factors, including variety, cultivation location and practices, season, and climate.^40,41^ FG mainly comprises carbohydrates (26–30%, with 1.5% of dietary fiber), proteins (1.5–2.1%), lipids (0.1–0.2%), sulfur compounds (1.1–3.5%), phenols (17.16–42.53 mg of gallic acid equivalent (GAE)/g), and more complex substances such as saponins (0.04–0.11%). It also contains vitamins (0.015%) and minerals (0.7%), such as C, E, B-group vitamins, calcium, sodium, potassium, magnesium, phosphorus, zinc, copper, iron, sulfur, manganese, and selenium.^42–45^

Fresh garlic is particularly rich in γ-glutamylcysteine, which undergoes hydrolysis and oxidation to form alliin. Actions like cutting, crushing, or chewing garlic can disrupt its cellular structure, resulting in the release of alliinase, an enzyme stored in vacuoles. This enzyme catalyzes the conversion of alliin into allicin, which imparts the characteristic pungent odor of garlic. This reaction also produces pyruvic acid as a byproduct. Allicin and other thiosulfinates are rapidly converted into several compounds, such as diallyl sulfide, diallyl disulfide, diallyl trisulfide, dithiins, and ajoene. Simultaneously, γ-glutamylcysteine is converted into SAC.^46,47^

The conversion from FG to BG induces substantial changes in its chemical profile (Table 1), which are influenced by processing conditions.

**Table 1:** Comparative Analysis of the Chemical Composition of Fresh and Black Garlic^a^

constituents		fresh garlic	black garlic	changes from FG to BG	references
macro-components	reducing sugars	292.54 ± 2.01 mg/100 g	754.51 ± 4.05–4726.04 ± 15.74 mg/100 g	↑	^44^
		1.52 ± 0.01 g/kg	2.73 ± 0.32–16.07 ± 0.38 g/kg		^5^
	proteins	10.62%	11.75%	↑	^5^
	amino acids	843.11 ± 3.75 mg/100 g	167.65 ± 1.08 mg/100 g	↓	^44^
	lipids	0.11%	0.43%	↑	^5^
micro-components	water-soluble vitamins	6632.91 ± 18.62 mg/kg	7618.24 ± 28.47–12,742.57 ± 28.09 mg/kg	↑	^51^
	minerals	1173.50 ± 2.43 mg/100 g	1337.71 ± 2.77–1358.94 ± 2.81 mg/100 g	↑	^44^
	organic acids	4.6 g/kg	33.61 ± 0.17–37.50 ± 0.17 g/kg	↑	^31^
	polyphenols	13.91 ± 1.62 mg GAE/g	25.81 ± 1.59–58.33 ± 1.90 mg GAE/g	↑	^48^
	melanoidins	negligible	0.12 ± 0.01–0.59 ± 0.01 μg/cm	↑	^4^
	β-carboline alkaloids	trace amounts	30-fold increase	↑	^54^
	allicin	3.45 g/kg	0.16 ± 0.07–0.93 ± 0.07 g/kg	↓	^31^
	SAC	19.61 ± 0.35 μg/g	28.78 ± 1.64–124.67 ± 1.61 μg/g	↑	^3^
	5-HMF	not detected	1.88 ± 0.02–4.82 ± 0.06 g/kg	↑	^31^
	pyruvic acid	486.71 ± 12.08 μmol/100 g	2456.54 ± 23.93 μmol/100 g	↑	^19^

a Data are presented as mean ± SD or %. Abbreviations: BG, black garlic; FG, fresh garlic; GAE, gallic acid equivalent; HMF, 5-hydroxymethylfurfural; SAC, S-allyl-l-cysteine.

During the ripening of black garlic, polysaccharides are degraded into oligosaccharides, disaccharides, and monosaccharides. Fructans progressively degrade under high-temperature conditions and the action of fructan exohydrolase. Specifically, Lu et al. highlighted that this phenomenon is largely attributable to the thermal treatment, while enzymatic hydrolysis plays a secondary role, as the enzyme is rapidly inactivated at the temperatures employed.^2^ Consequently, BG contains more reducing sugars than FG, imparting a sweeter taste to the final product.^44,48^ The content of these sugars also depends on their consumption during the Maillard reaction.^31^ The predominant reducing sugars in black garlic are fructose (57.14%), sucrose (7.62%), and glucose (6.78%).^49^

Furthermore, Nassur et al. observed a minor increase in protein content in BG compared to FG.^5^ Nevertheless, protein degradation may also occur from enzymatic or nonenzymatic hydrolysis, leading to an initial increment in amino acids content.^34^ Although the amino acid profile varies significantly depending on the ripening conditions, Kang documented a change in the total amount of 14 free amino acids from 843.11 ± 3.75 to 167.65 ± 1.08 mg/100 g of substance.^44^ An accumulation of certain amino acids – such as leucine, isoleucine, and phenylalanine – has been observed, accompanied by a reduction in others. In particular, the depletion of cysteine and tyrosine may be attributed to their involvement in the Maillard reaction.^48^ In conjunction with the degradation of hexoses in an acidic environment, this process contributes to the formation of HMF. Additionally, it produces melanoidins, which cause garlic browning.^31,50^ Kang reported a rise in the melanoidin content during the thermal process.^4^

In addition, an almost 4-fold increase in crude lipid content was observed when the bulbs were subjected to the aging process. Nonetheless, further studies are required to elucidate the changes in the lipid profile.^5^

The transformation of FG into BG also results in a 1.15- to 1.92-fold rise in water-soluble vitamin content. Nevertheless, thermal treatment under high-humidity conditions and increased acidity causes a reduction in certain vitamins. This includes thiamine (vitamin B1), biotin (vitamin B7), cobalamin (vitamin B12), vitamin C, and a wide array of fat-soluble vitamins. Conversely, an augmented concentration of niacin (vitamin B3) and pantothenic acid (vitamin B5) has been recorded. The former may be attributed to its release following the disruption of cell membranes, while the latter might arise due to the concentration effect resulting from reduced moisture content.^51^

This process also leads to a concomitant rise in the quantity of minerals, particularly sodium, potassium, iron, and calcium.^44^

Additionally, BG contains high levels of β-carboline alkaloids, which are derived from tryptophan. These compounds are only found in trace amounts in FG, yet during ripening 1,2,3,4-tetrahydro-β-carboline derivatives are formed, thus contributing to its antioxidant activity.^52,53^

As Zhang et al. observed, the organic acid content varies from 4.6 to 33.61, 37.50, 30.96, and 36.37 g/kg when FG is transformed into BG at 60 °C, 70 °C, 80 °C, and 90 °C, respectively.^31^ Particularly, levels of acetic and formic acids increase, which affects the flavor of garlic.^34^ Furthermore, Bae et al. reported a decrease in pH from 6.42 to 5.00 and 3.05 after exposing FG to 40 and 85 °C for 45 days.^3^

Among dietary vegetables, garlic is particularly rich in phenolic compounds, which are known for their antioxidant properties.^54,55^ The aging of FG into BG increases their concentration, enhancing its antioxidant activity.^32^ Choi et al. documented a rise in total polyphenols from 13.91 mg GAE/g to 25.81–58.33 mg GAE/g, depending on processing conditions.^48^ This may be attributed to the release of bound phenolics and enhanced extractability resulting from the disruption of cellular structures during thermal treatment.^56^ Kim et al. identified hydroxycinnamic acid derivatives as the primary phenolic acids in black garlic, with flavanols being the predominant flavonoid class.^32^ However, extended exposure to high temperatures can reduce certain phenolic compounds.^8^

In addition, pyruvate levels also increase during garlic ripening, contributing to BG antioxidant activity.^19^ This compound is typically produced by the alliin-allicin pathway.^47^

Furthermore, BG exhibits lower allicin levels than FG, as this compound is unstable and rapidly produces other organosulfur compounds. Allicin also reacts with l-cysteine to form S-allylmercaptocysteine.^57^

Lastly, thermal treatment leads to a 4- to 6-fold rise in S-allyl-l-cysteine content, depending on the temperature applied. Indeed, Bae et al. found that SAC reached 124.67 μg/g of dry matter when garlic was subjected to a temperature of 40 °C for 45 days, but dropped to 85.46 μg/g at 85 °C.^3^ This variation occurs since at lower temperatures (30–50 °C) SAC is primarily produced through the enzymatic hydrolysis of γ-glutamyl-S-allylcysteine (GSAC) by γ-glutamyl transpeptidase (GGT), whereas at higher temperatures SAC formation occurs through the nonenzymatic hydrolysis of GSAC and, to a lesser extent, by the reduction of alliin, as GGT becomes inactive under these conditions.^3,58^

An overview of the effects of the ripening process on black garlic quality attributes and chemical composition is provided in Table 2.

**Table 2:** Effects of the Ripening Process on Black Garlic Quality Attributes and Chemical Composition^a^

black garlic quality attributes/chemical composition	effects of the ripening process	references
color	changes from white to black due to melanoidin and HMF formation	^3,4,30,31^
texture	softer and chewy due to cell wall polysaccharide degradation and tissue softening under high temperature and controlled moisture conditions	^8,31^
flavor/odor	increase in reducing sugars → sweeter taste; increase in acetic and formic acids; conversion of allicin into SAC and SAMC → pungent odor reduced; bitter taste at high T (90 °C)	^1,8,31,34,44^
carbohydrates	degradation of polysaccharides into simple reducing sugars (fructose, sucrose, and glucose)	^2,44,48,49^
proteins/amino acids	minor protein content; altered amino acid profile (most relevant: ↓ cysteine and tyrosine, consumed in the Maillard reaction)	^5,34,44,48^
lipids	nearly 4-fold increase	^5^
vitamins	increase in water-soluble vitamins (B3 and B5); decrease in B1, B7, B12, C and fat-soluble vitamins	^51^
minerals	increase in sodium, potassium, iron, and calcium	^44^
sulfur compounds	allicin converted into SAC and SAMC; SAC increases 4–6 fold	^3,57,58^
polyphenols	increase in total polyphenols; hydroxycinnamic acid derivatives and flavanols are predominant; decrease with long high-T exposure	^8,32,48^
organic acids	increase (acetic and formic acids); pH decreases from 6.42 to 3.05 with T and time	^3,31,34^
other compounds	increase in β-carboline alkaloids, pyruvate, HMF, and melanoidins	^4,19,31,52,33^

a Abbreviations: HMF, 5-hydroxymethylfurfural; SAC, S-allyl-l-cysteine; SAMC, S-allylmercaptocysteine; T, temperature.

## 4 Biological Effects of Black Garlic

Black garlic and its derivatives exhibit a wide range of biological activities (Figure 3), primarily due to bioactive constituents that modulate key cellular signaling pathways. In recent decades, numerous preclinical studies have underscored the therapeutic potential of BG in the prevention and treatment of various diseases.

**Figure 3:**
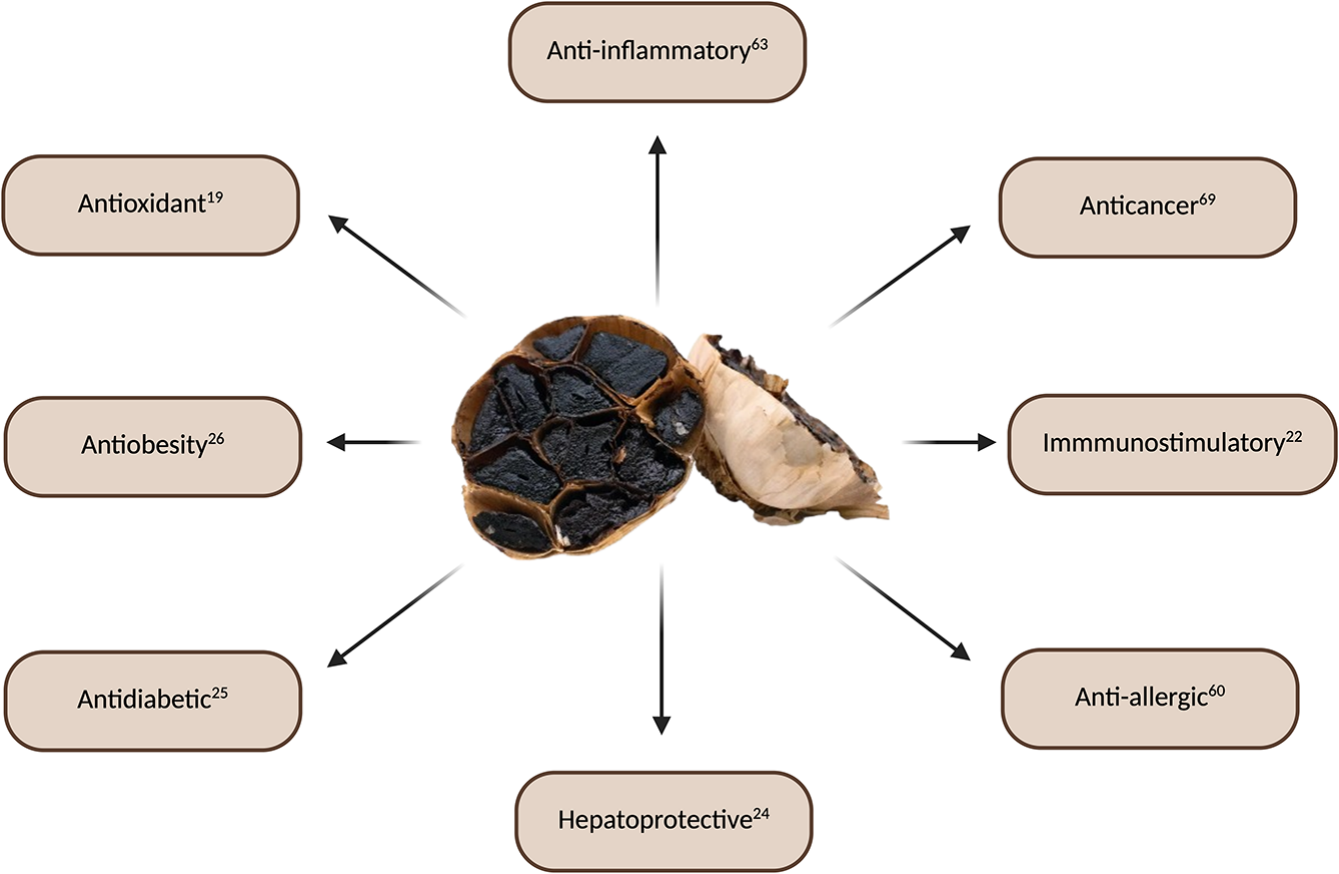
The principal biological effects of black garlic. Created in BioRender. BORGATTI, M. (2025) https://BioRender.com/gnxe36x.

### 4.1 Antioxidant Activity

In living organisms, oxidative processes naturally generate free radicals, including reactive oxygen species (ROS), which are typically neutralized by the antioxidant defense system. However, when free radical accumulation exceeds the capacity of these defenses, oxidative stress ensues, contributing to the development of chronic diseases such as cancer.^59^

Several constituents of black garlic have been shown to manifest substantial antioxidant activity. Jeong et al. highlighted the remarkable ability of BG to neutralize free radicals *in vitro* through 2,2-diphenyl-1-picrylhydrazyl and 2,2’-azinobis­(3-ethylbenzothiazoline-6-sulfonic acid) radical scavenging assays, with 50% inhibition (IC_50_) values of 166.3 ± 1.3 μg/mL and 108.1 ± 0.9 μg/mL, respectively. These values are approximately two- and 3-fold lower than those of the fresh garlic extracts.^19^ Additionally, at a dosage of 100 μg/mL, BG extracts maintain a strong capability to inhibit lipid peroxidation even after 5 days, whereas the same concentration of fresh garlic extracts showed a marked decline in inhibitory activity starting from day 3.^60^

Furthermore, experiments conducted on cell lines have demonstrated that BG extracts decrease hydrogen peroxide-induced ROS synthesis in murine macrophages RAW264.7 at 1000 μg/mL^19^ and counteract lipid peroxidation triggered by tert-butyl hydroperoxide in rat hepatocytes in a dose-dependent manner, at concentrations ranging from 2.5 to 10 μg/mL.^61^ In addition to *in vitro* studies, *in vivo* analyses have been conducted to evaluate the antioxidant properties of black garlic. Specifically, Lee et al. reported that BG consumption (administered through the diet, containing 5% of freeze-dried garlic or aged black garlic) significantly reduces hepatic levels of lipid peroxidation products in a murine model. This effect was more pronounced in the group treated with black garlic than in the one treated with fresh garlic. Simultaneously, an increase was observed in the activity of antioxidant enzymes such as superoxide dismutase (SOD), glutathione peroxidase (GPx), and catalase (CAT).^62^

### 4.2 Anti-inflammatory Activity

Among its multiple biological properties, black garlic also exhibits anti-inflammatory activity. In particular, 2-linoleoylglycerol isolated from BG (tested at concentrations between 5 and 20 μg/mL) has been shown to reduce the levels of nitric oxide (NO), prostaglandin E2 (PGE2), and inflammatory mediators such as interleukin (IL)-6, IL-1β, and tumor necrosis factor-alpha (TNF-α), as well as the expression of inducible nitric oxide synthase (iNOS) and cyclooxygenase-2 (COX-2), in lipopolysaccharide (LPS)-activated murine macrophages RAW264.7. This anti-inflammatory effect appears to be mediated through the inhibition of phosphorylation of mitogen-activated protein kinases (MAPK; MAPKs) extracellular signal-regulated kinase (ERK) and p38.^63^

Additional *in vitro* studies have demonstrated that 30 and 50 μg/mL of BG extract also suppresses cell cycle progression and proliferation in TNF-α-activated human endometrial stromal cells by inhibiting ERK and c-Jun N-terminal kinase activity. This process is accompanied by reduced expression of intracellular adhesion molecule-1 and vascular cell adhesion molecules-1, which may play a crucial role in the pathological process underlying endometriosis. Moreover, a decrease in IL-6 secretion was observed, likely due to inhibition of nuclear factor kappa B (NF-κB) and activator protein-1, two key transcription factors involved in inflammatory responses.^64^

*In vivo* studies have also revealed that BG extract (120 mg/kg, orally administrated) protects mice from LPS-induced septic shock by reducing the production of TNF-α and IL-6.^20^ These findings suggest that black garlic may serve as a supportive treatment for various inflammation-related conditions, including sepsis and endometriosis.

### 4.3 Anticancer Activity

Cancer remains one of the leading causes of mortality worldwide. Conventional treatments – such as surgery, chemotherapy, radiotherapy and hormonal therapy – are often associated with severe adverse effects that compromise patients quality of life.^65^ In recent years, researchers have begun to explore the integration of plant-derived natural compounds into conventional oncological regimens.^66^

Carcinogenesis is influenced by external and internal factors. Among internal contributors, excessive free radical accumulation constitutes a major etiological agent. Oxidative stress is frequently associated with chronic inflammation, which may induce genetic mutations in adjacent cells, leading to increased proliferation and the establishment of a microenvironment conducive to cancer development.^67^ Accordingly, the antioxidant and anti-inflammatory properties of black garlic may indirectly hinder cancer progression.^68^ In addition to these indirect mechanisms, BG appears to exert direct anticancer effects, as evidenced by numerous studies highlighting its antiproliferative, antimetastatic, and pro-apoptotic activities.^21,69,70^

Several *in vitro* experiments have shown that black garlic inhibits the proliferation of various cancer cell lines. For instance, BG extract has been found to suppress the growth of HT-29 human colon cancer cells in a time- (24, 48, and 72 h) and dose-dependent (20, 50, and 100 mg/mL) manner, and promote apoptosis by acting on the phosphoinositide 3-kinase/protein kinase B (Akt) signaling pathway.^71^ Similarly, black garlic extract induced apoptosis in SGC-7901 human gastric cancer cells and even reduced tumor mass in a murine model, starting from the lowest tested dose of 200 mg/kg (intraperitoneally administered).^70^

Low doses (4, 6, and 10 μg/mL) of hexane extracts derived from BG have also demonstrated pro-apoptotic activity in human leukemic U937 cells by upregulating death receptor 4 and Fas ligand, thereby activating the extrinsic apoptotic pathway. Concurrently, the extract increased the expression of the pro-apoptotic protein Bcl-2 associated X-protein (Bax) and decreased the levels of the antiapoptotic protein B-cell lymphoma 2 (Bcl-2), promoting the intrinsic apoptotic pathway.^72^

Recent evidence indicates that 100 mg/mL of BG extract inhibits proliferation, migration, invasion, and metastasis of MCF-7 and MDA-MB-361 human cells derived from breast adenocarcinoma. These effects are associated with downregulation of antiapoptotic proteins myeloid cell leukemia-1 (Mcl-1) and Bcl-2, and upregulation of pro-apoptotic proteins Bcl-2-interacting mediator of cell death (Bim) and Bcl-2 homologous antagonist/killer (Bak).^21^

Shin et al. demonstrated that 0.5 mg/mL of BG extract has the ability to impede the invasion and metastasis of AGS human gastric cancer cells through the suppression of matrix metalloproteinases 2 and 9. These enzymes play a pivotal role in the extracellular matrix degradation process, a critical step in the invasion of surrounding tissues by cancer cells and their subsequent dissemination to distant sites.^69,73^ Finally, Purev et al. reported a dose-dependent (100–500 μg/mL) cytotoxic effect of BG extract against multiple cancer cell lines, including lung carcinoma A549, gastric adenocarcinoma AGS, and hepatocarcinoma HepG2 cells.^22^

### 4.4 Immunostimulatory Activity

Black garlic has also been shown to act as an immunostimulant – a term that refers to substances capable of enhancing the immune system – making it potentially beneficial for the prevention and treatment of various diseases. Indeed, treatment with BG extract <100 μg/mL has been observed to increase human lymphocyte proliferation, as well as TNF-α release and NO production by macrophages. These immunostimulatory effects may also contribute to its anticancer activity.^22^

### 4.5 Antiallergic Activity

In recent years, the number of individuals affected by allergies has increased, prompting a growing interest in functional foods with potential antiallergic effects.^74^

Allergic responses are classified into four types based on the immune mechanisms involved. Type I hypersensitivity reaction is mediated by the activation of the high-affinity immunoglobulin E receptor, which is localized on the plasma membrane of mast and basophilic cells. Upon activation, this receptor triggers the release of mediators such as histamine, which are responsible for allergic symptoms. In addition, β-hexosaminidase – an established marker of cell degranulation – is released.^23^ Kim et al. demonstrated that BG extract inhibits β-hexosaminidase release in RBL-2H3 cells, a widely used *in vitro* model for studying allergic responses. However, when directly compared under identical conditions, FG extracts exerted a stronger inhibitory effect at both tested concentrations (10 and 100 μg/mL), suggesting that the transformation of fresh garlic into black garlic may reduce, at least in part, its antiallergic potential.^60^

Furthermore, an *in vivo* study showed that an ethyl acetate fraction of BG extract significantly reduced the cutaneous anaphylactic reaction in a murine model, but only at the highest tested dose of 66.7 mg/kg.^23^

### 4.6 Hepatoprotective Activity

Hepatic diseases represent a significant global health burden due to their high morbidity and mortality rates.^75^ Various substances, including dietary components, pharmaceuticals, alcohol, and pollutants, are known to induce both acute and chronic liver diseases.^76–79^ Despite advances in modern medicine, effective therapeutic strategies to stimulate hepatic function, protect the liver, or promote hepatic cell regeneration remain limited. Consequently, great attention has been directed toward identifying natural compounds capable of mitigating hepatic injury.^75^ In this context, some studies have demonstrated that black garlic also has hepatoprotective effects.^80,81^

For example, Shin et al. reported that chronic dietary administration of BG extract at 100 and 200 mg/kg for 4 weeks reduced aspartate aminotransferase and alanine aminotransferase levels – which are widely used markers of hepatocellular damage – in the liver of rats treated with carbon tetrachloride and d-galactosamine to cause liver injury, in a dose dependent manner. Similar results were observed in mice fed a high-fat diet.^24^

### 4.7 Antidiabetic Activity

Diabetes mellitus is a metabolic disorder characterized by chronic hyperglycemia and abnormalities in carbohydrate, fat, and protein metabolism due to defects in insulin secretion and/or action.^82^ Sustained hyperglycemia represents a major contributor to oxidative stress, with elevated levels of free radicals playing a crucial role in the pathogenesis and complications of diabetes.^83^

Within this context, the antidiabetic potential of black garlic and its effects on conventional diabetes markers have been investigated.^84,85^ For example, Kang et al. demonstrated that BG extract added to the diet (1% and 3% of the total food amount) of streptozotocin-induced diabetic rats reduced blood glucose and glycosylated hemoglobin levels.^25^

### 4.8 Antiobesity Activity

Obesity has emerged as a pressing public health concern and is associated with various diseases.^86^ In this framework, an *in vitro* study revealed that BG extract at 2 and 4 mg/mL suppresses adipogenesis by inhibiting key pro-adipogenic transcription factors in 3T3-L1 preadipocytes^87^ and, at similar doses, promotes lipolysis in mature adipocytes.^88^

Additionally, an *in vivo* study showed that BG administered in the diet at low (0.2%), medium (0.6%), and high (1.2%) concentrations reduces body weight, peritoneal fat, and serum triglycerides while improving hepatic lipid profiles (total lipids, triglycerides, and cholesterol) in rats with high-fat diet-induced obesity.^26^

Analogous findings were reported by Ha et al.,^89^ who observed a reduction in body weight and an improvement in lipid profile in rats fed a high-fat diet and supplemented with 0.5% or 1.5% of BG extract, compared to the control group. Specifically, in order to investigate the underlying mechanism, hepatic sterol regulatory element-binding protein 1c (SREBP-1c) mRNA expression was measured and found to be reduced following BG treatment.

SREBP-1c is a pivotal transcription factor that induces the expression of various genes involved in lipid metabolism, including acetyl-CoA carboxylase, fatty acid synthase, and glucose-6-phosphate dehydrogenase. Consequently, the levels of these enzymes were found to be significantly lower in the treated mice than in controls. This suggests that their inhibition contributes to a reduction in hepatic lipid synthesis. The mechanism in question was also associated with a decrease in plasma triglyceride levels. In addition, a reduction in the hepatic expression of hydroxymethylglutaryl-coenzyme A reductase and acyl-coenzyme A cholesterol acyltransferase was observed compared to the control group. This result was related to reduced total cholesterol levels. Conversely, an increase in high-density lipoprotein cholesterol – known for its protective effects on lipid metabolism – was reported.^89^

### 4.9 Additional Biological Effects

In addition to the biological activities previously discussed, black garlic has shown potential neuroprotective properties. These effects have been attributed to its ability to enhance spatial memory and increase the number of pyramidal cells in the hippocampus and Purkinje cells in the cerebellum of rats exposed to monosodium glutamate – a well-known neurotoxic compound – at all the tested doses (2.5, 5, and 10 mg/200 g of body weight).^90,91^

Furthermore, at 50 mg/mL black garlic has demonstrated antihypertensive activity by inhibiting the angiotensin-converting enzyme (ACE) *in vitro*.^92^ This enzyme catalyzes the conversion of angiotensin I into angiotensin II. The latter acts as a potent vasoconstrictor and stimulates aldosterone secretion from the adrenal cortex, leading to sodium retention in the kidneys and increased blood pressure.^93^

Emerging data also suggests that BG may have beneficial effects on gastrointestinal function. For example, Chen et al. reported that its administration at low (200 mg/mL) and high (400 mg/mL) doses improves intestinal motility in rats.^94^

## 5 Biological Activity of Single Compounds in Black Garlic

The health-promoting effects of black garlic are primarily attributed to its rich profile of bioactive compounds (Figure 4), which exert a broad spectrum of biological functions that have been investigated through both *in vitro* and *in vivo* studies (Table 3).

**Figure 4:**
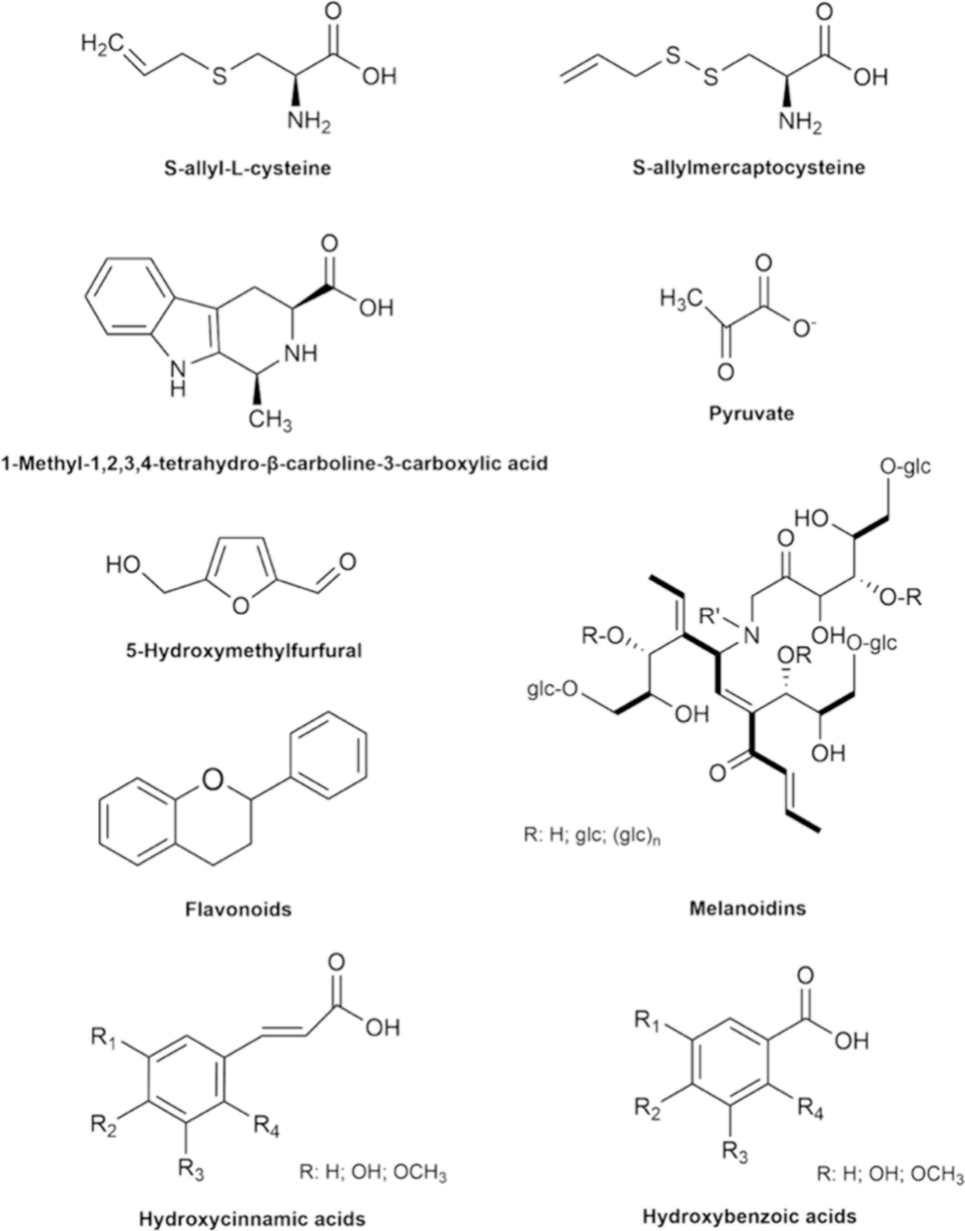
Chemical structures of key bioactive compounds identified in black garlic.

**Table 3:** Main Compounds in Black Garlic and Their Primary Biological Properties^a^

compounds	biological mechanisms (key biomarkers)
SAC	antioxidant activity (↓ROS; ↑CAT; ↑GPx; ↓MDA),^95,96,104^ carcinogen detoxification (↑GST),^97^ inhibition of cancer cell proliferation (↑CDK inhibitors; ↓CDK-activating kinase; ↓cyclin expression; ↓Rb-phosphorylation),^98^ induction of cancer cell apoptosis (↑caspase-3; ↑caspase-8; ↑caspase-9; ↑BAK; ↓Bcl-2),^98,99^ EMT and metastasis suppression (↑E-cadherin; ↓MMP-2; ↓VEGF),^100,101^ hypolipemic (↓cholesterol; ↓triglyceride),^104^ hypoglicemic (↑Insulin secretion; ↑SOD; ↑CAT; ↑GSH; ↓TBARS),^103^ neuroprotective (↓TBARS; MDA; ↑SOD; ↓ROS; ↓dopamine loss; ↓iNOS),^105,106^ hepatoprotective (↓apoptotic markers),^107^ and anti-inflammatory (↓inflammatory cytokines e.g. TNF-α, IL-β, and IL-6; ↓TLR4; ↓NLRP3; ↓NF-κB signaling pathway; ↓TNF-α; ↓iNOS; ↓COX-2)^108,109^ activities
SAMC	antioxidant (↓ROS/RNS; ↓4-HNE; ↓MDA; ↑SOD),^113,115,116^ hepatoprotective (↓p53; ↓apoptotic markers; ↑autophagy via mTOR inhibition),^114^ and anti-inflammatory (↓IL-1β; ↓IL-6; ↓TNF-α)^115^ activities, induction of cancer cell apoptosis (↑TGF-β signaling pathway; ↑Bax; ↑Bad; ↑Bim; ↑caspase-3; ↑caspase-9; ↓Bcl-2),^121,122^ inhibition of cancer cell proliferation (↑p53; ↑p21),^124^ and suppression of cell invasion (↑E-cadherin)^119^
1,2,3,4-tetrahydro-β-carboline derivatives	antioxidant activity (↓ROS)^130^
pyruvate	antioxidant (↓ROS),^19^ anti-inflammatory (↓NO; ↓PGE2),^19^ and neuroprotective (↓caspase-3)^133^ activities
HMF	antioxidant (↓ROS/RNS; ↓MDA; ↑CAT; ↑GPx; ↑SOD)^138^ and anti-inflammatory (↓NO; ↓PGE2; ↓iNOS; ↓COX-2; ↓inflammatory cytokines e.g. TNF-α, IL-1β, and IL-6; ↓VCAM-1; ↓ICAM-1; ↓NLRP3; ↓NF-κB, MAPK, and Akt/mTOR signaling pathways)^137,139,140^ activities, G_0_/G_1_ cell cycle arrest in cancer cells,^138^ and cancer cell apoptosis^138^
melanoidins	antihypertensive (ACE inhibition),^142^ antimicrobial (metal chelation),^144^ antiobesity (↓FAS; ↓LPL; ↑HSL),^145^ and antioxidant activity (↓lipid peroxyl radicals; metal chelation)^146^
polyphenols	antioxidant (↓ROS; metal chelation; ↑SOD; ↑GPx; ↑CAT; ↑α-tocopherol; ↓MDA; ↓NOX),^154–157^ prooxidant (↑ROS; ↑NOX; metal ions reduction),^177^ immunomodulatory and anti-inflammatory (↓inflammatory cytokines and chemokines e.g. TNF-α, IL-6, and IL-8; ↓NF-κB, MAPK, PI3K/Akt, mTOR, and JAK/STAT signaling pathway; ↓TLR; ↓PLA2; ↓LOX; ↓COX; ↓iNOS; ↓TREM-1),^29,156,166,167^ antimicrobial,^182^ antiviral (e.g., inhibition of HIV reverse transcriptase and integrase),^184^ antihypertensive (↑NO; ACE inhibition),^185^ antiobesity (↓lipogenesis; ↑lipolysis; ↑fatty acid β-oxidation; ↓triglyceride accumulation),^186^ and antidiabetic (↓blood glucose; ↑insulin secretion; ↓HbA1c; ↑PI3K/Akt signaling pathway)^190^ activities, inhibition of procarcinogens activation (↓CYPs),^171^ carcinogen detoxification (↑phase II enzymes e.g. GST),^171^ inhibition of cancer cell proliferation (↓cyclins; ↓CDKs; ↑p21; ↑p27; ↑p53; inhibition of topoisomerase II),^171,179^ suppression of angiogenesis and cancer cell invasion (↓VEGF; ↓HIF-α; ↓PDGF; ↓MMP-2; ↓MMP-9; ↑E-cadherin),^171,180^ induction of cancer cell apoptosis (↑Bax; ↓Bcl-2; ↑caspase-3; ↑caspase-9),^180^ epigenetic regulation (modulation of DNMTs, HMTs, HATs, and HDACs)^181^

a Abbreviations: 4-HNE, 4-hydroxy-2-nonenal; ACE, angiotensin-converting enzyme; Akt, protein kinase B; ATGL, adipose triacylglyceride lipase; Bad, Bcl-2-associated death promoter; Bax, Bcl-2 associated X-protein; Bcl-2, B-cell lymphoma 2; Bim, Bcl-2-interacting mediator of cell death; CAT, catalase; CDK, cyclin dependent kinase; COX, cyclooxygenase; CYPs, cytochrome P450; DNMTs, DNA methyltransferases; EMT, epithelial-mesenchymal transition; FAS, fatty acid synthase; GPx, glutathione peroxidase; GSH, glutathione; GST, glutathione S-transferase: HATs, histone acetyltransferases; HbA1c, hemoglobin A1c; HDACs, histone deacetylases; HIF-α, hypoxia inducible factor-alpha; HIV, immunodeficiency virus; HMF, 5-hydroxymethylfurfural; HSL, hormone sensitive lipase; ICAM-1, intercellular adhesion molecule-1; IL, interleukin; iNOS, inducible nitric oxide synthase; JAK, Janus kinase; LOX, lipoxygenase; MAPK, mitogen-activated protein kinase; MDA, malondialdehyde; MMP, metalloproteinase; mTOR, mammalian target of rapamycin; NF-κB, nuclear factor kappa-B; NLRP3, NLR family pyrin domain containing 3; NO, nitric oxide; NOX, NADPH oxidase; PDGF, platelet-derived growth factor; PGE2, prostaglandin E2; PI3K, phosphoinositide 3-kinase; PLA_2_, phospholipase A2; Rb, retinoblastoma protein; RNS, reactive nitrogen species; ROS, reactive oxygen species; SAC, S-allyl-l-cysteine; SAMC, S-allylmercaptocysteine; STAT, signal transducer and activator of transcription; TBARS, thiobarbituric acid reactive substances; TGF-β, transforming growth factor-beta; TLR, toll-like receptor; TNF-α, tumor necrosis factor-alpha; TREM-1, triggering receptor expressed on myeloid cells-1; VCAM-1, vascular cell adhesion molecule-1; VEGF, vascular endothelial growth factor.

### 5.1 *S*-Allyl-l-cysteine

Among the principal bioactive compounds in black garlic, S-allyl-l-cysteine has attracted particular interest. In fact, this sulfur-containing amino acid exhibits multiple biological activities. First of all, the antioxidant properties of SAC have been deeply investigated both *in vitro* and *in vivo*. The *in vitro* studies demonstrate its ability to scavenge ROS and hypochlorous acid, thereby protecting LLC-PK1 kidney cells from potassium dichromate-induced oxidative damage.^95^ The *in vivo* studies corroborated these findings by assessing the activities of the antioxidant enzymes SOD, CAT, and GPx: following the administration of SAC (150 mg/kg) for 45 days, diabetic Wistar rats exhibited enhanced activity of these enzymes in liver and kidney tissues.^96^ Beyond its antioxidant action, SAC also demonstrates anticancer activity *in vitro* through several mechanisms of action, such as the induction of carcinogen detoxification,^97^ inhibition of cell proliferation,^98^ induction of apoptosis,^98–100^ and suppression of epithelial-mesenchymal transition and invasion^100,101^ of cancer cells.

Similar evidence has been observed in animal models, where SAC consumption was shown to suppress the growth of lung carcinoma in xenografted BALB/CAnN-Foxn1 nude mice.^102^

Further *in vivo* studies extend these findings, highlighting how SAC is also effective in lowering blood glucose in streptozotocin-induced diabetic rats,^103^ reducing serum triglyceride and cholesterol levels,^104^ and exerting neuroprotection. Regarding this last aspect, it has been demonstrated that SAC reduces lipid peroxidation, ROS production, and dopamine loss in the striatum, thereby improving motor deficits in mice treated with 1-methyl-4-phenyl-1,2,3,6-tetrahydropyridine, a toxin used to induce Parkinson’s disease in animal models.^105^ Likewise, Ashafaq et al. demonstrated SAC’s ability to reduce oxidative damage and improve neurological deficits in a rat model of focal cerebral ischemia.^106^

S-allyl-l-cysteine also exhibits hepatoprotective properties. For example, it has been shown to protect BRL-3A rat liver cells against alcohol-induced apoptosis.^107^ Furthermore, SAC reduces the levels of pro-inflammatory cytokines such as IL-1β, IL-6, and TNF-α, demonstrating anti-inflammatory activity in mice.^108,109^ Additional reported benefits *in vivo* include nephroprotective,^110^ cardioprotective,^111^ and antihypertensive activities.^112^

### 5.2 *S*-Allylmercaptocysteine

S-allylmercaptocysteine is a water-soluble organosulfur compound with demonstrated antioxidant properties both *in vitro* and *in vivo*. Specifically, it scavenges hydroxyl radical and singlet oxygen, inhibits lipid peroxidation *in vitro*, and mitigates kidney damage in rats treated with gentamicin, an antibiotic known to induce nephrotoxicity via oxidative stress. These nephroprotective effects are associated with the prevention of decreases in antioxidant enzymes such as glutathione reductase and manganese superoxide dismutase.^113^

SAMC also exhibits hepatoprotective activity *in vivo*, as evidenced by its ability to protect the liver of rats affected by nonalcoholic fatty liver disease against chronic injury through inhibition of apoptosis and enhancement of autophagy.^114^

Its anti-inflammatory effects were demonstrated by Yang et al., who observed reduced levels of the pro-inflammatory cytokines IL-1β, IL-6, and TNF-α in the serum of mice treated with posaconazole, suggesting its capacity to attenuate this antifungal drug-adverse effects.^115^

The anticancer potential of SAMC has also been widely studied. It has been shown to reduce the onset and progression of various tumors through multiple mechanisms, *in vitro* and *in vivo*.^116,117^ For instance, SAMC prevents benzo­(a)­pyrene-induced carcinogenesis in human lung A549 cells by reducing ROS formation, increasing SOD activity, inhibiting NF-κB, suppressing cell proliferation, and regulating the cell cycle.^116^ Additionally, SAMC has also proven to be effective against cancer cells derived from multiple organs, including the colon, prostate, liver, breast, stomach, bladder, thyroid, and ovary.^117–124^ Finally, in xenografted mice SAMC could effectively suppress the growth and metastasis of colorectal cancer cells.^117^

### 5.3 1,2,3,4-Tetrahydro-β-carboline Derivatives

β-carboline alkaloids are known for their wide range of biological activities, including anticancer, antiviral, antimicrobial, antiparasitic, and anxiolytic effects.^125–128^ Among them, 1-methyl-1,2,3,4-tetrahydro-β-carboline-3-carboxylic acid (THβC) has been identified in black garlic. This compound likely forms during the ripening process through a condensation reaction between acetaldehyde – a byproduct of the Maillard reaction – and tryptophan.^53,129^ A salient property of THβC is its substantial antioxidant activity *in vitro*, which encompasses the scavenging of hydrogen peroxide and the inhibition of lipid peroxidation.^130^ To date, direct *in vivo* confirmation is still lacking.

### 5.4 Pyruvate

Pyruvate is abundant in black garlic and contributes significantly to its antioxidant properties. Indeed, it not only suppresses ROS generation but also reduces NO and PGE2 production induced by LPS in RAW264.7 cells. These findings suggest that pyruvate has anti-inflammatory effects. Also *in vivo,* multiple studies have demonstrated that exogenous pyruvate exerts diverse biological effects, including antioxidant,^131^ anti-inflammatory,^132^ and neuroprotective activities.^133^ However, the properties observed in black garlic appear to be less prominent than those exerted by pyruvate alone, indicating that other BG constituents might interfere with its activity.^19^

### 5.5 5-Hydroxymethylfurfural

5-Hydroxymethylfurfural is a furanic compound formed as an intermediate in the Maillard reaction.^134^ The process of formation is of pivotal significance in the characteristic color transition of garlic during thermal treatment. In particular, when HMF levels reach approximately 4 g/kg, BG acquires its distinctive dark appearance.^31^

Although it remains unclear whether HMF exposure poses a health risk, it seems that it possesses weak genotoxic and mutagenic potential only at high concentration.^135,136^ Despite these concerns, a mounting body of evidence suggests that HMF concurrently engenders multiple beneficial effects.^137^ For instance, Zhao et al. reported that HMF exhibits a strong antioxidant activity. It reduces ROS production and lipid peroxidation while enhancing the activity of the antioxidant enzymes GPx, SOD, and CAT in human erythrocytes treated with 2,2′-azobis­(2-amidinopropane) dihydrochloride, a compound employed to induce oxidative damage. These observations indicate a protective effect against oxidative stress *in vitro*.^138^

Furthermore, HMF also displays anti-inflammatory properties through the suppression of NO, PGE_2_, TNF-α, IL-1β, and IL-6 production in LPS-stimulated RAW264.7. In addition, it downregulates the expression of iNOS and COX-2, key mediators of inflammation. The anti-inflammatory effect of HMF appears to be mediated by the inhibition of the MAPK, NF-κB, and Akt/mammalian target of rapamycin (mTOR) signaling pathways.^139^

Additionally, HMF has demonstrated anticancer activity through G_0_/G_1_ phase arrest and induction of apoptosis, as evidenced by its antiproliferative effects on human melanoma A375 cells.^138^

However, *in vivo* validation is still limited and partly contradictory. For example, Zhang et al. demonstrated that the intraperitoneal injection of HMF in mouse models of acute-lung injury ameliorated disease conditions by exerting anti-inflammatory and protective effects.^140^ Conversely, a study conducted on Brown Norway rats highlighted the nonallergic anaphylaxis induced by HMF, underlying its related immunotoxic risks.^141^ However, most evidence remains restricted to cell-based analysis and further investigations are necessary to clarify these aspects.

### 5.6 Melanoidins

Melanoidins are heterogeneous, nitrogen-containing brown polymers. Similarly to 5-hydroxymethylfurfural, these pigments are synthesized during the final stages of the Maillard reaction and contribute to the characteristic dark color of thermally processed garlic.^4,30^

Beyond their role in color development, melanoidins have attracted considerable interest due to their diverse biological activities. Notably, melanoidins have exhibited antihypertensive properties, which are attributed to their capacity to inhibit ACE activity *in vitro*.^142^

Additionally, they have demonstrated antimicrobial effects against both Gram-positive (*Staphylococcus aureus* and *Listeria monocytogenes*)^142,143^ and Gram-negative (*Salmonella enteritis* and *Escherichia coli*)^142^ bacteria. Interestingly, melanoidins act as bacteriostatic agents at low concentrations and display bactericidal activity at higher doses.^144^

Furthermore, melanoidins derived from black garlic have shown promising antiobesity effects. *In vivo* studies have shown that melanoidin supplementation significantly reduces body weight and white adipose tissue accumulation, while also decreasing blood glucose levels and improving lipid profile.^145^

Finally, melanoidins exhibit significant antioxidant capacity, mainly through metal-chelating and radical-scavenging mechanisms demonstrated by *in vitro* studies.^146,147^

### 5.7 Polyphenols

Polyphenols are naturally occurring compounds derived from the secondary metabolism of plants, where they serve a critical function in mitigating various environmental stressors, including ultraviolet radiation and pathogen aggression.^148^ Structurally, these phytochemicals feature one or more aromatic rings substituted with hydroxyl groups.^32^

Polyphenols are broadly classified into two groups: flavonoids and nonflavonoids. Each class comprises several subcategories, defined by the number of phenolic units in their molecular structure, the nature of substituent groups, and/or the linkage type between phenolic units.

Flavonoids share a common diphenylpropane (C6–C3–C6) skeleton, consisting of two benzene rings connected by a three-carbon unit that typically forms an oxygen-containing heterocyclic ring. Variations in the hydroxylation pattern and the oxidation state of the central ring allow further classification into flavanols, anthocyanidins, isoflavones, flavones, flavonols, flavanones, flavanonols, neoflavonoids, and chalcones.^55,149^

In contrast, nonflavonoids generally exhibit simpler structures, often consisting of a single aromatic ring. This group includes phenolic acids, stilbenes, and lignans. Among these, phenolic acids represent the principal subgroup and are primarily derived from benzoic and cinnamic acids.^55^

Polyphenols are common constituents of plant-based foods and beverages, and their content is influenced by numerous factors such as environmental conditions, harvest ripeness, storage methods, and culinary processing.^150–152^ Garlic subjected to various thermal treatments has been found to contain significantly higher total polyphenol content compared to fresh garlic. According to Kim et al., flavanols (catechin, epicatechin, and epigallocatechin gallate) are the most abundant flavonoids in BG, followed by flavonols (myricetin, morin, and quercetin). Regarding phenolic acids, derivatives of hydroxycinnamic acid (caffeic acid, p-coumaric, m-coumaric, o-coumaric, and ferulic acid) are the most prevalent, although hydroxybenzoic acid derivatives (gallic and vanillic acid) have also been identified.^32^

The health-promoting potential of polyphenols is well-documented. Diets rich in polyphenol-containing foods are associated with a reduced incidence of chronic diseases, including cancer, cardiovascular disorders, and neurodegenerative conditions.^153^ Oxidative stress has been implicated as a common etiological factor among many of these diseases.^59^ Within this framework, polyphenols have demonstrated potent antioxidant properties by scavenging free radicals, acting as reducing agents, hydrogen donors, and singlet oxygen quenchers. Furthermore, they chelate transition metals such as ferrous ion (Fe^2+^), thereby preventing the formation of additional free radicals via the Fenton reaction, which occurs between Fe^2+^ and hydrogen peroxide. Moreover, polyphenols contribute to redox homeostasis by regenerating vitamin E.^152,154–156^ *In vivo* studies have shown that polyphenols also increase serum levels of antioxidant enzymes such as SOD, GPx, and CAT, while reducing lipid peroxidation.^157–159^

Neuroprotective effects have also been attributed to polyphenols, potentially reducing the incidence of Parkinson’s and delaying the onset of Alzheimer’s disease, primarily due to their antioxidant capabilities.^156,160–162^

Beyond their antioxidant activity, polyphenols exhibit notable immunomodulatory and anti-inflammatory effects, *in vitro* and *in vivo*. They influence immune cell populations, regulate cytokine production, and modulate the expression of pro-inflammatory genes.^156,163–165^ For instance, polyphenols interfere with the NF-κB and MAPK signaling pathways, reducing the formation of pro-inflammatory cytokines.^166,167^ They also modulate the expression and activity of cyclooxygenase and 5-lipoxygenase, leading to a reduction in the synthesis of prostaglandins and leukotrienes – two major mediators of inflammation.^29,168,169^

Due to this broad range of bioactivities, polyphenols have garnered increasing attention for their chemopreventive potential. Their protective effects stem primarily from their capacity to mitigate oxidative stress, a critical factor in carcinogenesis and cancer progression.^170^ Furthermore, they inhibit procarcinogens activation by reducing the activity of phase I metabolizing enzymes, while facilitating detoxification from carcinogenic substances through the induction of phase II metabolizing enzymes.^171^ These aspects were confirmed *in vivo*, as reported in a study on green tea polyphenols, which upregulated the expression of detoxifying enzymes such as heme oxygenase 1 and NAD­(P)H quinone oxidoreductase, while reducing transaminases and total bilirubin levels in the liver of Kunming mice.^172^

Beyond these detoxifying properties, polyphenols also influence epigenetic regulation, which is pivotal in cancer development as it modulates gene expression without altering the underlying DNA sequence. Specifically, they are capable of inhibiting DNA methyltransferases and histone deacetylases, as well as modulating histone acetyltransferases. This leads to the reactivation of tumor suppressor genes and the downregulation of oncogenes transcription *in vitro* ^173,174^ and *in vivo*.^175,176^

Although widely recognized for their antioxidant activity, polyphenols can also exhibit prooxidant effects under certain conditions, particularly at high concentrations, elevated pH, and in the presence of transition metals. Such behavior is attributed to the formation of an unstable aroxyl radical, which may react with oxygen to generate superoxide anion (O_2_^•‑^). Beyond direct ROS generation, some polyphenols promote oxidative stress by stimulating intracellular ROS production via NADPH oxidase or through the reduction of metal ions involved in redox-cycling.^177^

This dual redox behavior has particular relevance in the context of cancer. Compared to normal cells, cancer cells frequently display elevated oxidative stress and disrupted redox homeostasis. This imbalance has the potential to stimulate cell proliferation and activate adaptive responses that may contribute to tumorigenesis, metastasis, and treatment resistance. However, further exposure to ROS has been demonstrated to trigger cell death in cancer cells. Conversely, normal cells are typically less sensitive to ROS-inducing stimuli, as they maintain redox homeostasis through efficient adaptive mechanisms.^178^ Accordingly, the prooxidant activity of polyphenols may contribute to apoptosis induction and cell cycle arrest in cancer cells. In addition, they suppress specific signaling pathways involved in cell proliferation, which are typically hyperactivated during tumorigenesis.^156^

Further *in vitro* studies have indicated that some polyphenols also possess the ability to inhibit DNA replication, transcription, and repair in cancer cells.^179,180^ They also counteract angiogenesis by downregulating pro-angiogenic molecules such as vascular endothelial growth factor and exert antimetastatic effects through the suppression of metalloproteinase expression and the modulation of epithelial-to-mesenchymal transition.^156,181^

In another domain, polyphenols exhibit antimicrobial activity through multiple mechanisms, including disruption of bacterial membrane integrity and inhibition of certain enzymes. Although the exact pathways remain incompletely elucidated, it has been hypothesized that polyphenols can selectively induce the death of pathogenic species while promoting the growth of beneficial microorganisms.^182,183^ Some polyphenols have also demonstrated antiviral activity. For instance, epigallocatechin gallate exhibit activity against human immunodeficiency virus, influenza virus, and hepatitis C virus.^184^

Phenolic compounds are additionally recognized for their potential to reduce the risk of cardiovascular disease, particularly through their antihypertensive properties. These include the enhancement of NO-mediated vasodilation, inhibition of ACE, and attenuation of oxidative stress.^185^

Furthermore, polyphenols have demonstrated antiobesity effects by decreasing lipogenesis, suppressing triglyceride accumulation, promoting lipolysis, and stimulating fatty acid β-oxidation.^186^ Multiple studies conducted on animal models and human subjects, in fact, highlight how these compounds reduce multiple obesity-related parameters, including the adipose tissue weight and the fat accumulation.^187–189^

In addition, a growing body of evidence from both *in vitro* and *in vivo* studies supports their role in the prevention and management of type 2 diabetes. Indeed, polyphenols enhance insulin secretion and sensitivity, thereby improving glycemic control.^190^

In light of this compelling evidence, it can be concluded that polyphenols represent an extremely diversified array of bioactive molecules with extensive health-promoting effects. Many of these biological properties are consistent with those attributed to black garlic, further supporting its relevance as a potential functional food.

## 6 Future Perspectives

The health benefits of black garlic have been extensively documented. However, the specific bioactive constituents responsible for its various biological effects, as well as the molecular mechanisms underlying their activity, remain only partially understood. Furthermore, the potential synergistic interactions among these compounds have received limited attention. Hence, further research is necessary to elucidate the contribution of individual key constituents and their potential interactions.

## Abbreviations

4-HNE: 4-hydroxy-2-nonenal
ACE: angiotensin-converting enzyme
Akt: protein kinase B
ATGL: adipose triacylglyceride lipase
Bad: Bcl-2-associated death promoter
Bak: Bcl-2 homologous antagonist/killer
Bax: Bcl-2 associated X-protein
Bcl-2: B-cell lymphoma 2
BG: black garlic
Bim: Bcl-2-interacting mediator of cell death
CAT: catalase
CDK: cyclin dipendent kinase
COX-2: cyclooxygenase-2
CYPs: cytochrome P450
DNMTs: DNA methyltransferases
EMT: epithelial-mesenchymal transition
ERK: extracellular signal-regulated kinase
FAS: fatty acid synthase
Fe2^+^: ferrous ion
FG: fresh garlic
GGT: γ-glutamyl transpeptidase
GPx: glutathione peroxidase
GSAC: γ-glutamyl-S-allylcysteine
GSH: glutathione
GST: glutathione S-transferase
HATs: histone acetyltransferases
HbA1c: hemoglobin A1c
HDACs: histone deacetylases
HIF-α: hypoxia inducible factor-alpha
HIV: immunodeficiency virus
HMF: 5-hydroxymethylfurfural
HSL: hormone sensitive lipase
ICAM-1: intercellular adhesion molecule-1
IL: interleukin
iNOS: inducible nitric oxide synthase
JAK: Janus kinase
LOX: lipoxygenase
LPS: lipopolysaccharide
MAPK: mitogen-activated protein kinase
Mcl-1: myeloid cell leukemia-1
MDA: malondialdehyde
MMP: metalloproteinase
mTOR: mammalian target of rapamycin
NF-κB: nuclear factor kappa B
NLRP3: NLR family pyrin domain containing 3
NO: nitric oxide
NOX: NADPH oxidase
O2•-: superoxide anion
PDGF: platelet-derived growth factor
PGE2: prostaglandin E2
PI3K: phosphoinositide 3-kinase
PLA2: phospholipase A2
Rb: retinoblastoma protein
RNS: reactive nitrogen species
ROS: reactive oxygen species
SAC: S-allyl-l-cysteine
SAMC: S-allylmercaptocysteine
SOD: superoxide dismutase
SREBP-1c: sterol regulatory element-binding protein 1c
STAT: signal transducer and activator of transcription
T: temperature
TBARS: thiobarbituric acid reactive substances
TGF-β: transforming growth factor-beta
THβC: 1-methyl-1,2,3,4-tetrahydro-β-carboline-3-carboxylic acid
TLR: toll-like receptor
TNF-α: tumor necrosis factor-alpha
TREM-1: triggering receptor expressed on myeloid cells-1
VCAM-1: vascular cell adhesion molecule-1
VEGF: vascular endothelial growth factor.
